# Experimental Autoimmune Encephalomyelitis Development Is Aggravated by *Candida albicans* Infection

**DOI:** 10.1155/2015/635052

**Published:** 2015-04-19

**Authors:** Thais F. C. Fraga-Silva, Luiza A. N. Mimura, Camila M. Marchetti, Fernanda Chiuso-Minicucci, Thais G. D. França, Sofia F. G. Zorzella-Pezavento, James Venturini, Maria S. P. Arruda, Alexandrina Sartori

**Affiliations:** ^1^Department of Microbiology and Immunology, Biosciences Institute, São Paulo State University (UNESP), 18618-000 Botucatu, SP, Brazil; ^2^Department of Biological Sciences, School of Sciences, São Paulo State University (UNESP), 17033-360 Bauru, SP, Brazil

## Abstract

Multiple sclerosis (MS) is an inflammatory/autoimmune disease of the central nervous system (CNS) mainly mediated by myelin specific T cells. It is widely believed that environmental factors, including fungal infections, contribute to disease induction or evolution. Even though *Candida* infection among MS patients has been described, the participation of this fungus in this pathology is not clear. The purpose of this work was to evaluate the effect of a *Candida albicans* infection on experimental autoimmune encephalomyelitis (EAE) that is a widely accepted model to study MS. Female C57BL/6 mice were infected with *C. albicans* and 3 days later, animals were submitted to EAE induction by immunization with myelin oligodendrocyte glycoprotein. Previous infection increased the clinical score and also the body weight loss. EAE aggravation was associated with expansion of peripheral CD4^+^ T cells and production of high levels of TNF-*α*, IFN-*γ* IL-6, and IL-17 by spleen and CNS cells. In addition to yeast and hyphae, fungus specific T cells were found in the CNS. These findings suggest that *C. albicans* infection before EAE induction aggravates EAE, and possibly MS, mainly by CNS dissemination and local induction of encephalitogenic cytokines. Peripheral production of encephalitogenic cytokines could also contribute to disease aggravation.

## 1. Introduction

Multiple sclerosis (MS) is an inflammatory/autoimmune and demyelinating disease of the central nervous system (CNS). It is considered one of the most common neurological disorders and causes of disability in young adults [1]. The estimated number of people with MS has increased from 2.1 million in 2008 to 2.3 million in 2013 [2]. Animal models, particularly experimental autoimmune encephalomyelitis (EAE), have been essential to decipher the pathophysiology of MS [3–6]. MS and EAE are characterized by an autoimmune response against CNS proteins, mediated mainly by T cells, that culminates in inflammatory infiltrate, gliosis, damage of myelin sheath, and neuronal death [7–9].

This disease is thought to be triggered by a complex interaction between genetic and environmental factors. Expressive data confirm that genetic variation is an important determinant for MS risk. Population, family, and molecular studies strongly support a polygenic model of inheritance, driven primarily by allelic variants relatively common in the general population. The major histocompatibility complex is believed to be the strongest MS susceptibility locus genome-wide and was identified in all studied populations [10]. It has also long been recognized that infections may serve as environmental triggers for this disease. A large number of pathogens, including worldwide distributed fungi, have been proposed to be associated with MS [11]. As most of the systemic fungal pathogens have been associated with dissemination to the CNS [12], they could contribute to local tissue destruction by their presence or, alternatively, by the induction of a local immune response.

*Candida* spp. is one of these pathogens that could contribute to MS development.* C. albicans* is a pleomorphic fungus that colonizes the majority of healthy human individuals. This fungus can behave as a normal component of the microbiota and also as an opportunistic pathogen that causes superficial mucosal infections as well as disseminated disease [13, 14]. As the fourth most common cause of nosocomial infections,* C. albicans* is commonly isolated from immunocompromised individuals, including those with HIV, those immunosuppressed due to cancer treatment, and premature babies [15]. A possible association between MS and* Candida* spp. has been suggested by serological evidences. A significantly higher level of* Candida* specific antibodies was detected in MS patients than in normal control individuals [16]. In addition,* Candida* spp. antigens were also demonstrated in the cerebrospinal fluid of some MS patients [17].

The possible contribution of* Candida* spp. to MS pathogenesis was initially attributed to cross-reactivity with human tissues, including brain structures [18]. More recently, it was proposed that* Candida*, sequestered in nonneuronal tissues, could release toxins that would destroy astrocytes and oligodendrocytes generating myelin debris that would then trigger a pathogenic immune response in the CNS [19]. Furthermore, the presence of yeast and hyphae in the brain recruits inflammatory cells and elicits expansion of microglia cells [20]. Considering that the possible contribution of* C. albicans* to MS needs to be investigated and that elucidation of this could affect the treatment of this disease, we evaluated the possible deleterious effect of a previous* C. albicans* infection on EAE development.

## 2. Methods

### 2.1. Animals

Female C57BL/6 mice 9–11 weeks old were purchased from University of São Paulo (USP) (Ribeirão Preto, SP, Brazil). The animals received sterilized food and water* ad libitum* and were manipulated in accordance with the local Ethics Committee for Animal Experimentation (CEEA), São Paulo State University (UNESP) (Botucatu, SP, Brazil; protocol number 351).

### 2.2. EAE Induction

MOG35–55 peptide (MEVGWYRSPFSRVVHLYRNGK) was synthesized by Genemed Synthesis Inc. (San Antonio, Texas, USA). Mice were immunized subcutaneously with 100 *μ*g of MOG35–55 peptide emulsified in 25 *μ*L of Complete Freund's Adjuvant (CFA) containing 4 mg/mL of* Mycobacterium tuberculosis*. Mice also received 2 intraperitoneal doses, 0 and 48 hours after immunization, of 200 ng of* Bordetella pertussis* toxin (Sigma-Aldrich Corporation, St. Louis, MO, USA). EAE clinical assessment was daily performed according to the following criteria: 0, no symptoms; 1, limp tail; 2, hind legs weakness; 3, partially paralyzed hind legs; 4, complete hind leg paralysis; and 5, complete paralysis/death. The % of weight loss and the maximum clinical score were calculated considering the highest body weight loss and the highest clinical score that each animal reached during the experiment, independently of the period, and the result was expressed as the mean per experimental group.

### 2.3. Fungi

*C. albicans* strain FCF 14 (Genbank Accession EF591020) was originally obtained from the mycology collection of the Faculdade de Odontologia de São José dos Campos, UNESP, and maintained in our mycological collection on Sabouraud-dextrose agar (Difco Laboratories, Detroit, MI, USA). For mice infection,* C. albicans* was cultured on solid media during 24 hours at 37°C. The fungal concentration was adjusted to 5.0 × 10^7^/mL viable yeast cells in sterile saline solution (SSS). Fungus suspension was then inoculated into the lateral tail vein (0.1 mL/animal).

### 2.4. Fungal Load Determination

Samples from spleen, kidney, liver, brain, and spinal cord were weighted and macerated in 1.0 mL of SSS. Afterwards, 0.1 mL from each tissue homogenate was spread over culture plates containing Sabouraud-dextrose agar using a Drigalski T loop. The procedures were performed in duplicate. The plates were then sealed and incubated at 37°C for 3 days. The number of colony forming units (CFU) was normalized per gram of tissue.

### 2.5. CNS-Mononuclear Cells Isolation

Fourteen days after EAE induction, mice were anesthetized with ketamine/xylazine and perfused with 10 mL of SSS. Brain and spinal cord were collected, macerated, and digested with 2.5 mg/mL of collagenase D (Roche Applied Science, Indianapolis, IN, USA) in 4 mL of RPMI (Sigma) at 37°C for 45 min. Then, suspensions were washed in RPMI and centrifuged at 450 ×g at 4°C for 15 min. Cells were resuspended in Percoll (Sigma) 37% and gently laid over Percoll 70% in tubes of 15 mL. The tubes were centrifuged at 950 ×g for 20 min with centrifuge breaks turned off. After centrifugation the ring containing mononuclear cells was collected, washed in RPMI, and centrifuged at 450 ×g for 10 min. Cells were then resuspended in complete RPMI medium (RPMI supplemented with 10% of fetal bovine serum), counted, and analyzed.

### 2.6. Cell Culture Conditions and Cytokine Quantification

Spleen and CNS-isolated cells were collected and adjusted to 5 × 10^6^ cells/mL and 2 × 10^5^ cells/mL, respectively, in complete RPMI medium. Spleen and CNS-isolated cells were plated and stimulated with MOG (20 *μ*g/mL and 50 *μ*g/mL, resp.) and with* C. albicans* (5 yeasts/1 cell). Cytokine levels were evaluated 48 h later by enzyme-linked immunosorbent assay (ELISA) in culture supernatants using IFN-*γ* BD OptEIA Sets (Becton, Dickinson and Company, BD, Franklin, San Diego, CA, USA) and IL-2, IL-4, IL-6, IL-10, IL-17, and TNF-*α* Duosets (R&D Systems, Minneapolis, MN, USA). The assays were performed according to the manufacturer's instructions.

### 2.7. FACS Analysis

Spleen cells were collected; the red blood cells were lysed with buffer containing NH_4_Cl, and adjusted to 10^6^ cells/tube. CNS-extracted cells were plated at 5 × 10^5^ cells/well and stimulated with MOG (125 *μ*g/mL) and with* C. albicans* (5 yeasts/1 cell). After incubation at 37°C for 48 h, cells were collected and stained. Spleen and CNS-extracted cells were blocked with rat serum 1% for 20 min to prevent nonspecific binding via Fc receptor. After Fc blocking, cells were stained with 0.2 *μ*g of PerCP-conjugated anti-mouse CD3 and 0.25 *μ*g of FITC-conjugated anti-mouse CD4 for 20 min at 4°C. Intracellular FoxP3 transcription factor analysis was performed only in spleen samples by using CD3-PercP, CD4-FITC plus 0.13 *μ*g of APC-conjugated anti-mouse CD25 and 0.2 *μ*g of PE-conjugated anti-mouse FoxP3 and staining set (eBiosciences, San Diego, CA, USA) according to manufacturer's instructions. After staining, the cells were washed, resuspended in FACS buffer, and fixed in paraformaldehyde 1%. Analysis was performed using a FACSCanto II (BD) from Bioscience Institute (Botucatu, SP, Brazil) and the data were analyzed with FlowJo software (TreeStar, Ashland, OR, USA).

### 2.8. Histopathology of the CNS

After euthanasia, brain and lumbar spinal cord samples were removed and fixed in 10% neutral buffered formalin. Paraffin slides with 4 *μ*m were stained with hematoxylin and eosin (H&E) to evaluate the inflammatory process. A semiquantitative analysis of CNS inflammation was performed according to the following criteria: (0) inflammatory infiltration absent; (+/++) mild/moderate inflammatory infiltration; (+++) intense inflammatory infiltration. Sections were also stained with periodic acid-Schiff to visualize fungal structures.

### 2.9. Statistical Analysis

Results were expressed as mean ± standard deviation or with median and interquartile (25–75%) ranges. To test for the normality of data, results were analyzed by Shapiro-Wilk's test. Comparisons between two samples were made by *t*-test and more than three samples were made by one way ANOVA followed by Tukey's test for parametric variables and by Kruskal-Wallis followed by Dunn's test for nonparametric variables. Fisher's test was performed to estimate the frequency of* C. albicans-*positive tissues and to compare the semiquantitative analysis of CNS tissue inflammation. The data were analyzed using SigmaPlot statistical package for Windows version 2.0 (1995, Jandel Corporation, CA, USA) and values of *P* < 0.05 were considered statistically significant.

## 3. Results

### 3.1. *Candida albicans* Infection Disseminates to the CNS

**Figure 1:**
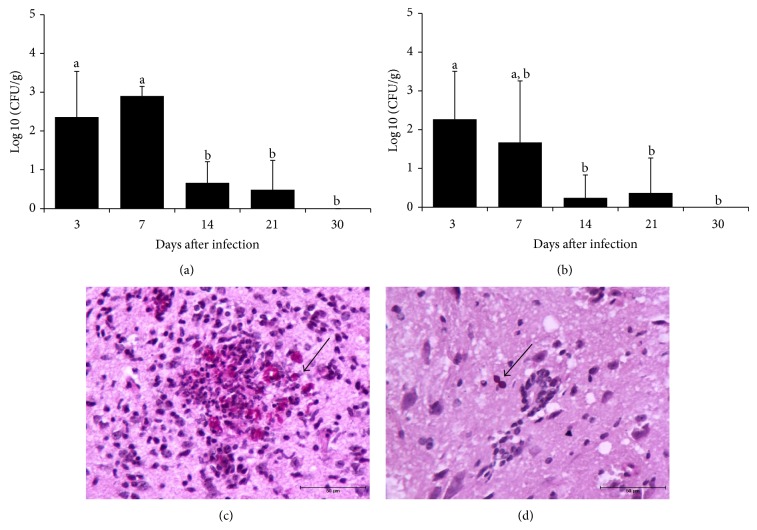
Dissemination of* C. albicans* to the central nervous system. C57BL/6 mice were infected with* C. albicans* and fungal load was evaluated 3, 7, 14, 21, and 30 days after in the brain (a) and in the spinal cord (b). The results are expressed as mean ± SEM (*n* = 5-6 mice/group) of the CFU (log⁡10) per gram of tissue. ANOVA, Tukey's test, *P* < 0.05. Different letters indicate statistical difference among the experimental time points. Periodic acid-Schiff revealed yeasts and hyphae in brain (c) and yeast in cervical spinal cord (d) sections.

We initially tested the characteristics of the* C. albicans* infection in C57BL/6 mice as this is one of the strains that are susceptible to EAE induction. Experimental infection with* C. albicans* in C57BL/6 mice determined a disseminated infection that also reached the CNS. As observed in Table 1, the viable fungi were recovered from all evaluated organs, including the brain and the spinal cord. After 30 days all organs, except the spleen, exhibited fungal clearance. The kinetics of fungal load, during 30 days, is showed in Figures 1(a) and 1(b) for brain and spinal cord, respectively, and indicates that the fungus load is more accentuated in the first week of infection. The presence of yeasts and hyphae in the brain and yeast in the spinal cord is illustrated in Figures 1(c) and 1(d), respectively.

### 3.2. Production of Potentially Encephalitogenic Cytokines during* C. albicans* Infection

**Figure 2:**
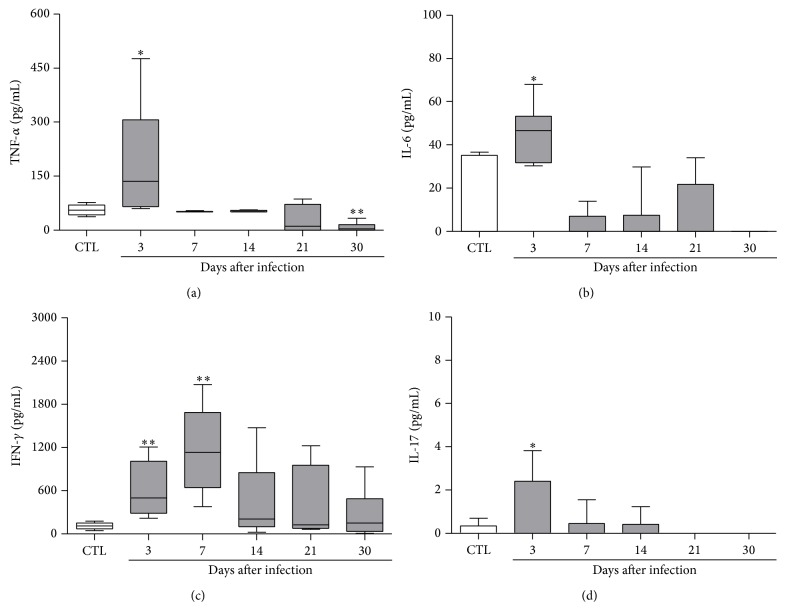
Kinetics of cytokine production by spleen cells from mice infected with* C. albicans*. C57BL/6 mice were inoculated with* C. albicans* and the spontaneous production of cytokines by spleen cells was evaluated 3, 7, 14, 21, and 30 days after fungal inoculation. The results are expressed as median, 25–75% (box), and minimum-maximum (error bars) of 5-6 mice/group. Mann-Whitney test, ^∗^ *P* < 0.05 and ^∗∗^ *P* < 0.01 indicate statistical difference between each experimental time point and the control group (uninfected).

As many of the most encephalitogenic cytokines are also involved in the defense against* C. albicans* and other fungi, we tested their production during the time periods when the fungus was being detected. Spleen cell cultures from infected mice produced elevated levels of TNF-*α*, IL-6, IFN-*γ*, and IL-17 (Figure 2). Cytokine levels were especially elevated in the 3rd day after infection.

### 3.3. Infection with* C. albicans* Aggravates EAE Development

**Figure 3:**
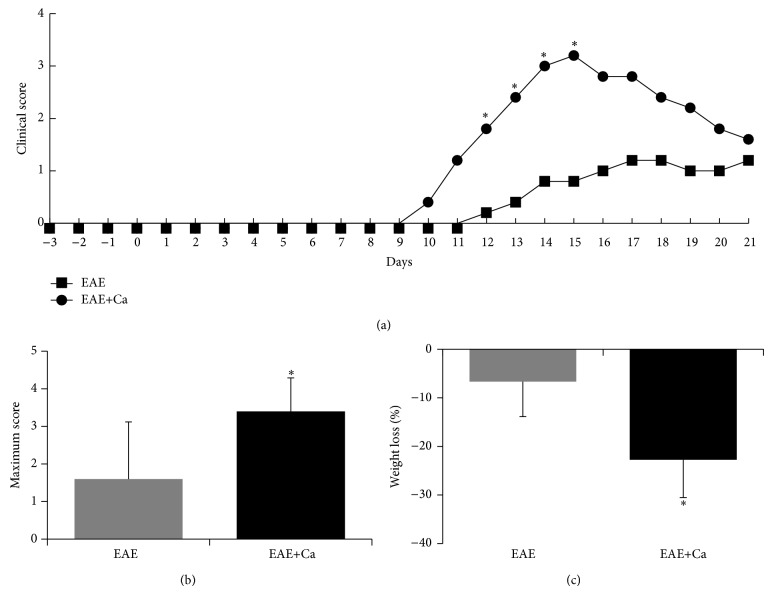
Effect of* C. albicans* on EAE development. C57BL/6 mice were infected with* C. albicans* 3 days before EAE induction. Disease development was followed during 21 days. Clinical scores (a) were checked every day and are expressed as mean; maximum clinical score (b) and % of body weight loss (c) were calculated as described in Methods section. The results (a and b) are expressed as mean ± SD (*n* = 6–8 mice/group). Unpaired *t* test, ^∗^ *P* < 0.05 indicates difference between EAE and EAE+Ca groups.

To test the possible deleterious role of* C. albicans* on EAE development, EAE was induced in mice that had been infected three days before with the fungus. Mice previously infected, denominated EAE+Ca group, developed a more severe form of encephalomyelitis. As shown in Figure 3(a), these animals already showed paralysis signs at the 9th day after EAE induction whereas the EAE control group presented paralysis only 2 days later. This higher disease severity was detected during the whole acute disease phase. The average maximum clinical score, as depicted in Figure 3(b), confirmed this worst clinical evolution. Weight loss was also more accentuated in this experimental group as can be observed in Figure 3(c).

### 3.4. Peripheral Immunological Alterations during EAE Aggravation by* C. albicans* Infection

**Figure 4:**
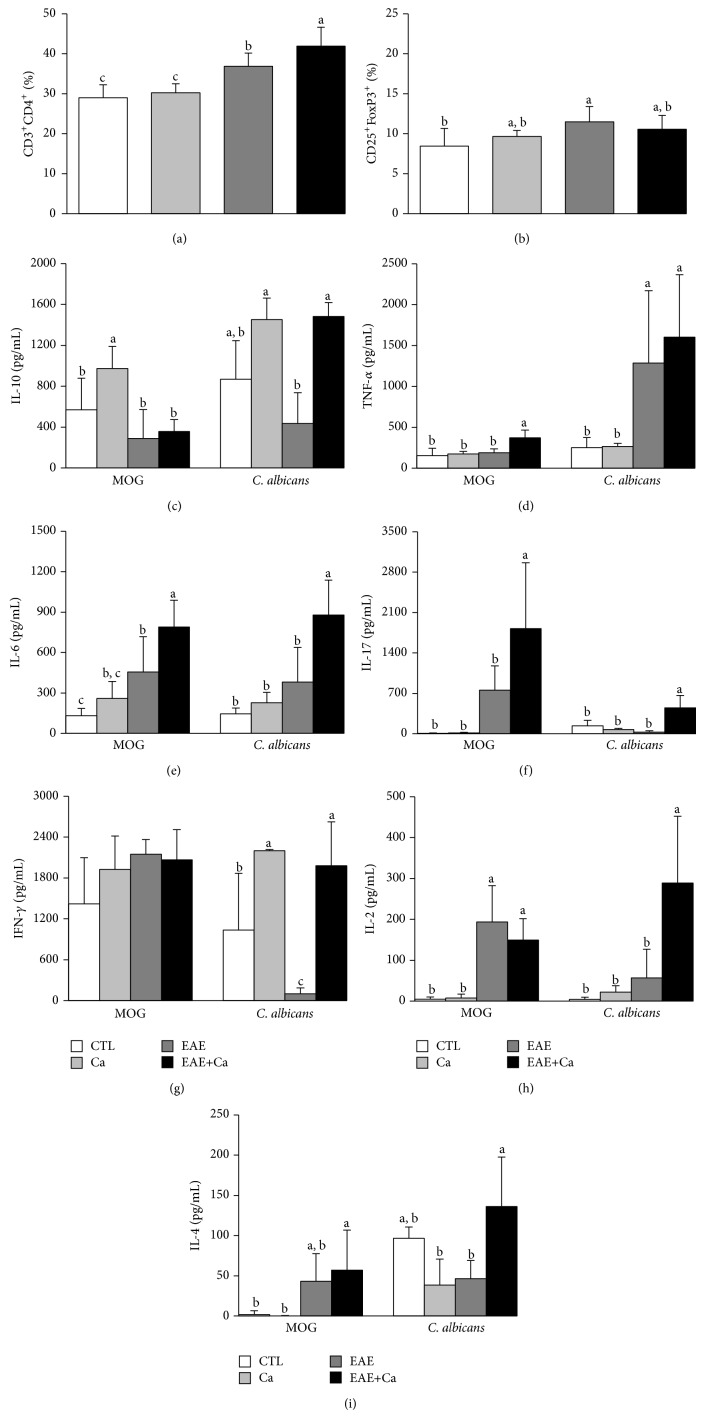
Modulation of MOG-induced cytokine production by previous infection with* C. albicans*. C57BL/6 mice were infected with* C. albicans* and 3 days later they were submitted to EAE induction. Fourteen days after EAE induction, some immunological parameters were evaluated in the spleen. The percentage of CD3^+^CD4^+^ (a) and CD3^+^CD4^+^CD25^+^FoxP3^+^ (b) was performed by cytometric analysis in 100.000 acquired events. IL-10 (c), TNF-*α* (d), IL-6 (e), IL-17 (f), IFN-*γ* (g), IL-2 (h), and IL-4 (i) levels were measured in spleen cell cultures stimulated with MOG or heat-killed* C. albicans*. The results are expressed as mean ± SD (*n* = 6–8 mice/group). ANOVA, Tukey's test, and *P* < 0.05. Different letters indicate statistical difference among the groups (a and b) or among the groups under the same* in vitro* stimulation (c, d, e, f, g, h, and i).

To evaluate if peripheral immunological parameters could explain this detrimental fungal effect on EAE, we tested the % of CD3^+^CD4^+^ and CD3^+^CD4^+^CD25^+^ FoxP3^+^ T-cell subsets. The cytokine production by spleen cells restimulated with MOG or with heat-killed* C. albicans* yeasts was also determined. Normal mice and mice only infected were also analyzed. A higher percentage of CD3^+^CD4^+^ T cells were found in EAE+Ca and EAE groups in comparison to normal and infected groups. In addition, the % of this T-cell subset was significantly higher in the group that was previously infected with the fungus (EAE+Ca) in comparison to the EAE group (Figure 4(a)). The % of the FoxP3^+^ T cells was significantly higher in the EAE, but not in the Ca and EAE+Ca groups, in comparison to the control group, as illustrated in Figure 4(b). Concerning cytokines induced by MOG, the EAE+Ca group presented a significant production of TNF-*α* (Figure 4(d)), IL-6 (Figure 4(e)), and IL-17 (Figure 4(f)) in comparison to all other experimental groups. IL-2 (Figure 4(h)) and IL-4 (Figure 4(i)) were similarly elevated in EAE and EAE+Ca groups. These two groups also produced low and similar amounts of IL-10 (Figure 4(c)). Comparison of EAE+Ca and EAE cytokine production induced by heat-killed* C. albicans* clearly showed that IL-10, IL-6, IL-17, IFN-*γ*, IL-2, and IL-4 were significantly higher in the previously infected group.

### 3.5. Local Immunological Alterations during EAE Aggravation by* C. albicans* Infection

**Figure 5:**
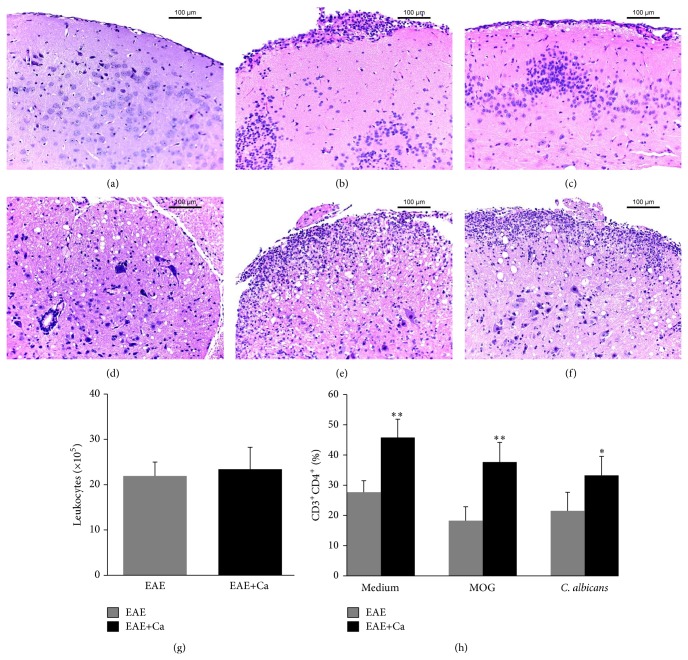
Previous infection with* C. albicans* increases the amount of CD4^+^ T cells in the CNS. C57BL/6 mice were infected with* C. albicans* and 3 days later they were submitted to EAE induction. Fourteen days after EAE induction, inflammation and % of CD4^+^ T cells were evaluated in the CNS. Inflammatory infiltrates detected by H&E staining are shown in brain samples from EAE (b) and from EAE+Ca (c) groups and in spinal cord samples from EAE (e) and from EAE+Ca (f) groups. A brain and spinal cord samples from a normal mouse is shown in (a) and (d), respectively. Total leukocyte number (g) and percentage of CD3^+^CD4^+^ T-cell subset (h) (analysis performed in 50.000 acquired events). The results are expressed as mean ± SD (*n* = 6-7 mice/group). Unpaired *t* test, ^∗^ *P* < 0.05 and ^∗∗^ *P* < 0.01 indicate difference between EAE and EAE+Ca groups under the same* in vitro* stimulation.

**Figure 6:**
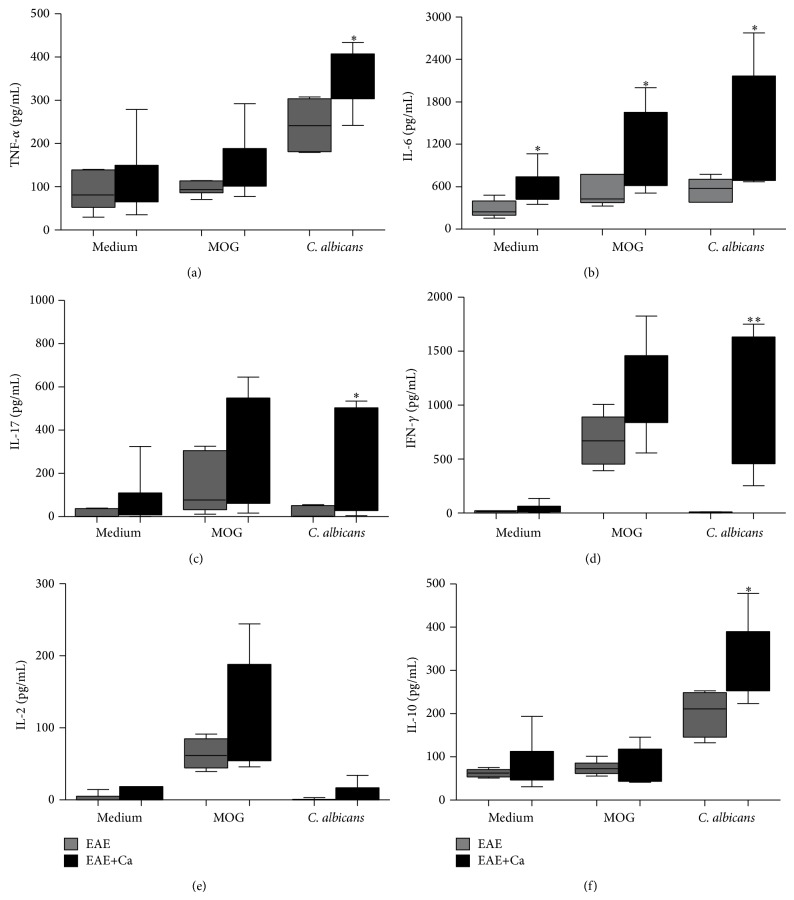
*Candida* specific T cells contribute to elevated production of encephalitogenic cytokines in the CNS. CNS eluted cells were restimulated with MOG or heat-killed* C. albicans* and TNF-*α* (a), IL-6 (b), IL-17 (c), IFN-*γ* (d), IL-2 (e), and IL-10 (f) levels were measured by ELISA. The results are expressed as median, 25–75% (box), and minimum-maximum (error bars) of 6 to 7 mice/group. Mann-Whitney test, ^∗^ *P* < 0.05 and ^∗∗^ *P* < 0.01 indicate statistical difference between EAE and EAE+Ca groups under the same* in vitro* stimulation.

H&E staining clearly indicated a strong and similar inflammatory process in the brain and spinal cord of both EAE and EAE+Ca animals, as shown in Figure 5. This analogous inflammatory process was confirmed by a semiquantitative analysis done in both brain and spinal cord samples (data not shown). As expected, no inflammatory infiltrates were present in normal mice (Figures 5(a) and 5(d)). The amount of total leukocytes eluted from the CNS from both experimental groups was also similar as depicted in Figure 5(g). The percentage of CD3^+^CD4^+^ T cells was always higher in the EAE+Ca group, independently of their previous stimulation with MOG or heat-killed* C. albicans* yeasts (Figure 5(h)). Cells eluted from the CNS of both groups respond in a similar way to* in vitro* stimulation with MOG, that is, they produced similar amounts of TNF-*α*, IL-17, IFN-*γ*, IL-2, and IL-10 (Figure 6). However, cells eluted from mice previously infected with* C. albicans* (EAE+Ca group) produced much more TNF-*α*, IL-6, IL-17, IFN-*γ*, and IL-10 in response to* C. albicans in vitro* restimulation (Figure 6).

## 4. Discussion

Multiple sclerosis (MS) is one of the world's most common neurological disorders [2]. The disease develops as a result of interactions between the environment and the immune system in genetically susceptible individuals and it has long been recognized that infections may serve as environmental triggers for MS [11]. Even though viral agents have been more usually suspected as aggravating or triggering agents of this disease, fungi, especially their toxins, were recently incriminated as relevant underlying causes of MS and thus may offer an approach towards a more effective adjunct treatment [19].* C. albicans* is the most common fungal pathogen of humans and its spreading to the brain has been described during acute infections [21, 22]. Interestingly, fifty percent of patients with disseminated candidiasis underwent CNS fungal invasion [23]. Even though* C. albicans* is usually more prevalent in immunocompromised individuals, it has also been reported to cause meningoencephalitis in healthy individuals [24]. Considering these aspects and the fact that a possible relationship between* Candida* spp. and MS patients [16, 17, 19] was recently described, we evaluated the effect of an experimental infection with this fungus on the development of EAE, which is a largely accepted model to study the pathophysiological mechanisms of MS [25].

We initially evaluated the characteristics of* C. albicans* infection in C57BL/6 mice, which is one of the strains that develop encephalomyelitis upon immunization with antigens from the CNS [26]. This strain developed a widespread infection characterized by involvement of the majority of the organs, including the brain and the spinal cord. This diffuse infection was, however, very well controlled by the immune system since almost no fungi were recovered after 30 days of infection. This dissemination of* C. albicans* to the brain was already demonstrated not only in C57BL/6 mice [20] but also in other mouse strains as BALB/c [27] and Swiss [28]. However, this is the first report that indicates spreading of this fungus to the spinal cord portion of the CNS in mice.

As expected, the infectious process triggered by* C. albicans* induced an elevated production of inflammatory cytokines as TNF-*α*, IL-6, IFN-*γ*, and mainly IL-17. This proinflammatory environment was more pronounced by the 3rd day of infection. As these cytokines have been clearly associated with MS and EAE due to their encephalitogenic properties [29–32], we choose this period of infection to induce EAE. This choice was also based on the fact that the fungus had already reached the CNS at this early time. C57BL/6 mice were then infected with* C. albicans* by intravenous route and 3 days later they were submitted to EAE induction. A very clear deleterious effect was observed in EAE development. The animals became sick earlier and, in addition, developed a more severe disease. Higher severity was characterized by both a higher body weight loss and a more accentuated degree of paralysis. To the best of our knowledge, this is the first demonstration that a previous experimental infection with* C. albicans* triggered EAE exacerbation. These findings are relevant because a direct contribution of* C. albicans* to this neurological disease has not been deeply investigated. However, a series of indirect and epidemiological findings supports this possibility. For example, Purzycki and Shain [19] proposed that certain pathogenic fungi could release toxins that, by destroying CNS astrocytes and oligodendrocytes, would degrade myelin triggering the onset of MS and its associated symptoms. By using immunofluorescence analysis, Benito-León et al. [16] suggested a serological evidence of a link between* Candida* infection and MS condition. By comparing the amount of anti-*Candida* antibodies in the sera of normal subjects and MS patients, these authors suggested that infections with* Candida* spp. could be associated with increased odds of MS [16]. In addition to specific antibodies, fungal macromolecules such as proteins, polysaccharides, and DNA were also detected in blood samples from MS patients [33]. Besides these serologic evidences, antibodies against* Candida* spp. [33] and fungal DNA [17] were also detected in the cerebrospinal fluid of MS patients.

To unravel, at least partially, the immunological mechanism involved in this effect, some peripheral immunological parameters were compared among EAE, EAE+Ca,* C. albicans* infected (Ca), and normal (CTL) experimental groups. Even though T regulatory (Treg) mediated responses remain poorly understood in* Candida* infection, data indicate increased proportion of this subset during candidiasis [34, 35]. As FoxP3^+^ T cells are mostly responsible for EAE recovery in C57BL/6 mice [36, 37], we initially hypothesized that Treg expansion could theoretically downregulate EAE development. This assumption was based on the fact that Treg cells induced during infectious diseases can regulate EAE in an apparently nonspecific manner [38]. To test this possibility we evaluated the effect of the* C. albicans* infection on the percentage of this T-cell subset. The expected increase in the percentage of FoxP3^+^ T cells was found in the spleen of the EAE group. However, the proportion of this T-cell subset was not modified in Ca and in EAE+Ca groups. This finding can be attributed, at least partially, to the complex relationship, including cell plasticity, between Treg and Th17 responses during* C. albicans* infection [35]. In addition to Treg cells we also evaluated the percentage of CD4^+^ T cells and cytokine production. Previous fungal infection increased CD4^+^ T-cell subset in spleen of EAE-mice (EAE+Ca group) and clearly upmodulated the production of many encephalitogenic cytokines by spleen cells stimulated with MOG or heat-killed* C. albicans*. Even though the effect of EAE on fungal load was not the focus of this investigation, fungi recovery was usually significantly lower in the infected animals that had also EAE (not shown). This finding suggests that the immune response against MOG, or maybe the presence of the CFA, is increasing fungicidal activity of the immune system. The higher production of encephalitogenic cytokines by both stimuli, MOG and* C. albicans*, was interpreted as a possible cause of EAE increased severity as cytokines can easily cross the blood-brain barrier and directly affect CNS functions [39, 40].

As the histopathology analysis from brain and spinal cord sections suggested similar degrees of inflammation, we compared the amounts of leukocytes and CD3^+^CD4^+^ T cells eluted from the CNS. Confirming the H&E analysis, this comparison revealed the presence of similar numbers of total cells in EAE and EAE+Ca groups, demonstrating therefore that the higher disease severity was not due to a higher degree of inflammatory infiltration. Nevertheless, the immunophenotyping analysis showed a higher proportion of CD3^+^CD4^+^ T-cell population in the EAE+Ca group. Culture of the cells eluted from the CNS showed, as expected, that they produced proinflammatory cytokines in the presence of MOG. Interestingly, they also produced significant amounts of proinflammatory cytokines when stimulated with* C. albicans*.

Together, these results are suggesting that both peripheral and local fungus effects are contributing to a more severe disease development. The translation of these findings to human patients certainly requires much more investigation in this area. However, we believe that these findings add more evidence that* C. albicans* is one of the fungi that can affect this type of neurological pathology.

## Figures and Tables

**Table 1:** Frequency of *C. albicans*-positive tissues.

Period	Tissue				
	Spleen	Kidney	Liver	Brain	Spinal cord
3 days	6/6	6/6	5/6	5/6	5/6
7 days	5/5	5/5	3/5	5/5	3/5
14 days	4/6	2/6	0/6	4/6	1/6
21 days	4/6	1/6	0/6	2/6	1/6
30 days	2/5	0/6	0/6	0/6	0/5

*P* value	0.0606	0.0022	0.0152	0.0152	0.0152

Data were expressed as number of *C. albicans*-positive animals/total number of animals per group.
